# Miscellaneous Facts and Remarks

**Published:** 1799-08

**Authors:** 


### Miscellaneous Facts and Remarks.

We have the satisfaction to announce to our readers, that the experiments lately made on the external use of tartarized antimony, by Mr. Hutchinson (of which we have taken notice in a preceding part of this work), are are fully confirmed by ſeveral of our medical friends, who have preſcribed a ſolution of emetic tartar, in the ſame proportion as directed by Mr. H. On wetting the palms of the hands with a ſponge dipped in the ſolution: drying them at the fire, and repeating the lotion, or friction, the effect was almoſt immediate, ſo that the patient ſoon fell into a profound ſleep.

As the beneficial and extenſive conſequences that are likely to reſult from this important diſcovery, muſt intereſt every medical practitioner, on account of the ſuperior advantages this remedy poſſeſſes, over the *internal* uſe of opiates, which ſo frequently are productive of miſchief, in plethoric and irritable individuals, as well as in every inſtance where the primæ viæ are either already loaded with, or yet liable to obſtructions,—we earneſtly ſolicit farther communications on this intereſting ſubject. We hope that our obliging correſpondents will direct their attention principally to thoſe caſes of mania, incipient phthiſis, hypochondriaſis, hyſteries, &c. where the nervous ſyſtem is, as it were, unhinged, inſomuch that the introduction of medicines by the ſtomach appears to be counteracted, and their uſual effects fruſtrated, by the irregular action, or preternatural re-action of the nerves.

---

In the '*Journal der Erfindungen, &c.*' No. 6, we meet with a letter from Dr. Consbruch, of Bielefeld, who aſſures us that, in *fiſtulous ulcers*, ariſing from a ſcrofulous cauſe, he cannot recommend a remedy more effectual, and affording more permanent relief, than the common red ſnail of the gardens, (*Helix pomatia Linn.*) externally applied alive, to the ulcerated part, and repeated once a day. The Doctor mentions three caſes in which the ſnail thus uſed was attended with the moſt deſirable effects, by healing ulcers of that deſcription in the courſe of ten days or a fortnight: he alſo ſtates that this ſimple remedy, has long been employed by the common people of Germany, with the beſt ſucceſs, in a ſimilar manner, externally, on tetters and other ſcorbutic eruptions. The manner in which the healing powers of the ſnail operate on external ulcerations, Dr. Conſbruch, endeavours to explain from the gentle ſtimulating, agglutinating properties of animal jelly; and he farther obſerves that ſimilar remedies have formerly been recommended by phyſicians in various diſeaſes of the ſkin, by directing patients to uſe a decoction of calve's-feet.

---

In the "*Annales de Chimie*," No. 89. p. 214, M. Heber, of Berlin, affirms, that he has been enabled to obtain a very efficacious tincture of antimony, by mixing with alcohol the liquid tartar, digeſted in vitrified antimony. To this article the French editor ſubjoins the following curious remark: "When we ſee remedies ſo violent, and at the ſame time ſo uncertain in their preparation, daily reproduced under new forms, and admitted into the Materi Medica, we cannot form a very favourable opinion of the philoſophy which has hitherto enlightened that ſcience."

---

Brugnatelli, the Italian chemiſt, remarks in the ſame Journal, No. 86, that he has for ſome time employed, with great ſucceſs, the acidulated carbonate of lime, in calculous affections.

MEDICAL

